# Spatially explicit density and its determinants for Asiatic lions in the Gir forests

**DOI:** 10.1371/journal.pone.0228374

**Published:** 2020-02-19

**Authors:** Keshab Gogoi, Ujjwal Kumar, Kausik Banerjee, Yadvendradev V. Jhala

**Affiliations:** Wildlife Institute of India, Chandrabani, Dehradun, Uttarakhand, India; Centre for Cellular and Molecular Biology, INDIA

## Abstract

Asiatic lions (*Panthera leo persica*) are an icon of conservation success, yet their status is inferred from total counts that cannot account for detection bias and double counts. With an effort of 4,797 km in 725 km^2^ of western Gir Protected Area, India, we used polygon search based spatially explicit capture recapture framework to estimate lion density. Using vibrissae patterns and permanent body marks we identified 67 lions from 368 lion sightings. We conducted distance sampling on 35 transects with an effort of 101.5 km to estimate spatial prey density using generalized additive modeling (GAM). Subsequently, we modeled lion spatial density with prey, habitat characteristics, anthropogenic factors and distance to baiting sites. Lion density (>1-year-old lions) was estimated at 8.53 (SE 1.05) /100 km^2^ with lionesses having smaller movement parameter (σ = 2.55 km; SE 0.12) compared to males (σ = 5.32 km; SE 0.33). Detection corrected sex ratio (female:male lions) was 1.14 (SE 0.02). Chital (*Axis axis*) was the most abundant ungulate with a density of 63.29 (SE 10.14) as determined by conventional distance sampling (CDS) and 58.17 (SE 22.17)/km^2^ with density surface modeling (DSM), followed by sambar (*Rusa unicolor*) at 3.84 (SE 1.07) and 4.73 (SE 1.48)/km^2^ estimated by CDS and DSM respectively. Spatial lion density was best explained by proximity to baiting sites and flat valley habitat but not as much by prey density. We demonstrate a scientifically robust approach to estimate lion abundance, that due to its spatial context, can be useful for management of habitat and human-lion interface. We recommend this method for lion population assessment across their range. High lion densities in western Gir were correlated with baiting. The management practice of attracting lions for tourism can perturb natural lion densities, disrupt behavior, lion social dynamics and have detrimental effects on local prey densities.

## Introduction

Conservation management of any endangered species depends on information of its population extent, abundance, and current threats [1]. Spatial density is of primary interest to ecologists and wildlife managers alike because of its decisive influence on ecological interactions, behavioral attributes, and site-specific human-wildlife conflict management [2–4]. Monitoring large carnivore populations with robust scientific methodology is essential because of their umbrella status [5], their charismatic persona that intrigues societies and political interest [6], and in many cases their endangered nature that requires unbiased estimates [7] for resource allocation and conservation management [8]. However, inappropriate monitoring methodology can result in biased estimates that subsequently lead to incorrect policy and management decisions that may be counterproductive for conservation [9]. Therefore, use of robust methods that provide unbiased and precise estimates to underpin correct management and policy decisions are an essential part of applied ecology [10].

The recovery of the last Asiatic lions (*Panthera leo persica*) in the Gir Forests of Gujarat, India from less than fifty individuals [11] to the current population claims of over 500 [12,13] is a modern conservation success story [14,15]. The lion population has in recent times extended its range from the Gir Protected Area (PA) [about 1,883km^2^] to cover between 7000 to 13,000 km^2^ of human dominated agro-pastoral landscape of Saurashtra [12,13]. However, the traditional total count method is used to estimate their abundance and status every five years by the Gujarat State Forest Department [16]. Total counts are rarely possible in a free ranging population since not all animals are detected and often it is not possible to avoid double counts of the same individuals. These shortfalls of total counts are explicitly addressed with a robust scientific approach to estimate abundance through individual animal identification and techniques such a capture-mark-recapture (CMR) [17] which accounts for imperfect detection. Individual identification of lion is possible from vibrissae patterns and permanent body marks [18,19]. The down-listing of Asiatic lions from critically endangered to endangered category by the IUCN [1,20] is based on assessment of their status done through total counts and can have implications on the conservation of the sub-species [9]. The methods for estimating endangered carnivores need to be robust yet practical, site-specific, cost effective and easily replicable. Capture-mark-recapture [21] was adopted to obtain more reliable estimates of lion abundance in parts of Gir [22,23]. However, CMR, though a substantial improvement over total counts, does not provide any spatial context to the abundance of the species and it estimates density ($\hat{\text{D}}$) based on a buffer of ½ MMDM (mean maximum distance moved by individual lions) as an *ad-hoc* consideration of effectively sampled area [24,25]. While spatially explicit capture recapture (SECR) models detections in space and computes density considering a spatial point process [24,26].

Herein, we demonstrate the use of the polygon search method in an SECR framework [27,28] to estimate spatial density of lions in the western part of the Gir PA. We simultaneously estimate prey density through transect based distance sampling using the density surface modelling (DSM) framework [29]. Subsequently, we evaluate the relationship of spatial lion density with prey, food provisioning to lions for tourism (baiting sites) and habitat characteristics. Our results show that current management practices, catering towards reducing conflict and enhancing lion viewing for tourism by baiting them had profound effect on lion spatial density and artificially inflated local densities. We discuss the impact of such practices on the social organization of lions and their prey.

## Methods and materials

### Ethics statement

All permissions to carry out the field research were obtained from the Chief Wildlife Warden, Gujarat State, under the provisions of the Wildlife (Protection) Act, 1972. The research comprised the masters dissertation of KG under the long-term study of Asiatic lion ecology by the Wildlife Institute of India. It was reviewed and approved by a committee constituted by the Dean and selected faculty members of the Wildlife Institute of India. This committee also considered the ethical aspects of the research.

### Study area

**Fig 1:**
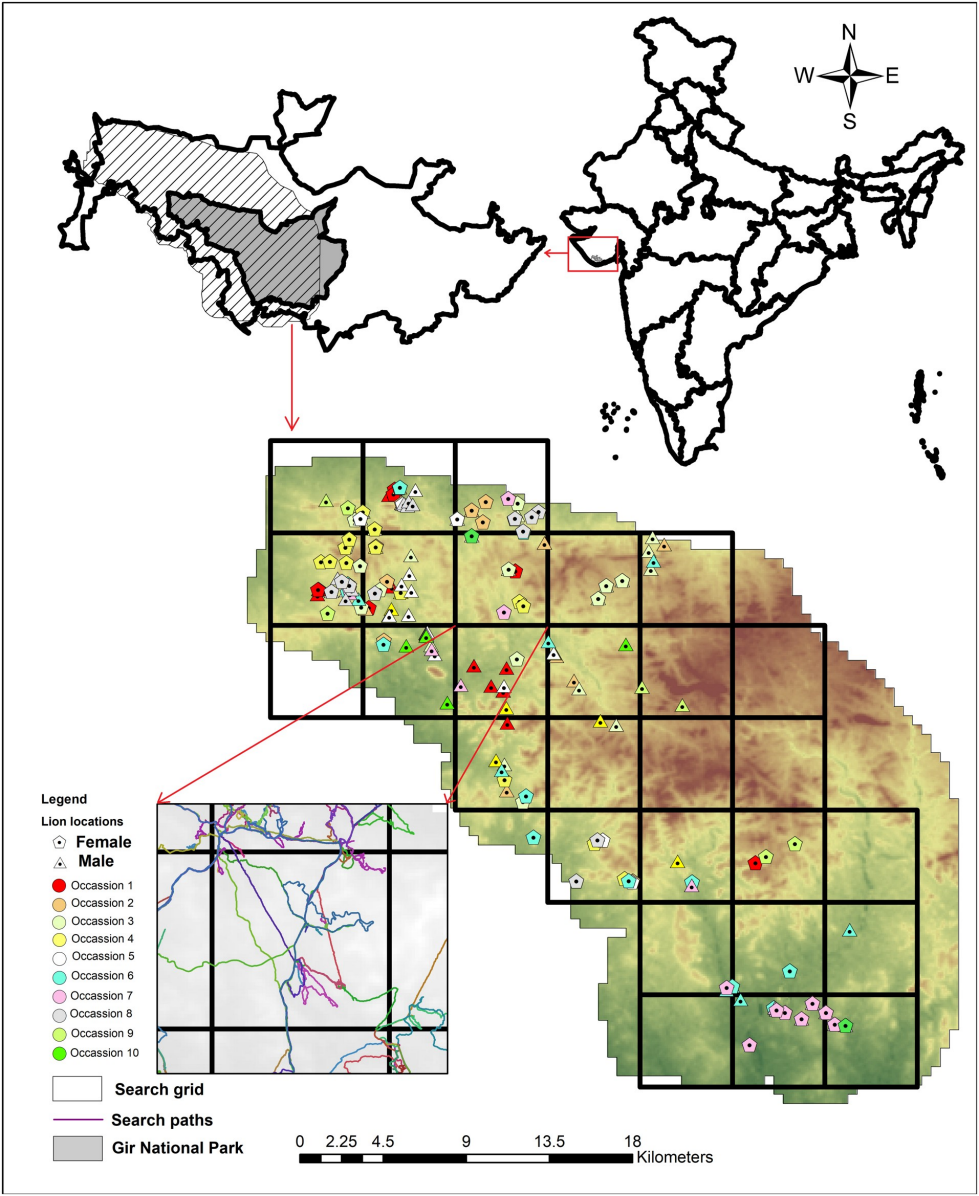
Study area within western and central parts of the Gir Protected Area. The map insets show the location of the study area within India and Gir Protected Area, wherein the National Park area was devoid of tourism. The enlarged study grid shows the search paths for lions on different sampling occasions.

Gir PA extending over an area of 1,883 km^2^ from 21° 20’ N to 20° 57’ N latitude to 70° 27’ E 71° 13’ E longitude [30]; (Fig 1) is a dry deciduous forest [31] situated in Gujarat province, western India and is made up of a Sanctuary (with human settlements, regulated livestock grazing, wildlife and religious tourism and other rights) covering 1,153 km^2^, a 259 km^2^ National Park (inviolate area devoid of any human habitation or use) and 471 km^2^ of additional reserve, protected and unclassified forests [32] (Fig 1). Gir has a semi-arid climate with an average annual temperature ranging from an average minimum of 5° C (winter) to an average maximum of 44 ° C (summer) and an average annual rainfall of 980 mm [32]. Rugged hilly terrain (elevation ranging from 83 m above msl to 648 m above msl) forms the catchment of seven perennial rivers. Dominant vegetation includes *Tectona grandis*, *Anogeissus* spp, *Acacia* spp and *Ziziphus* spp [33].

Apart from the last free-ranging population of the Asiatic lion, other carnivores found in Gir PA are leopard (*Panthera pardus*), striped hyena (*Hyaena hyaena*), golden jackal (*Canis aureus*), ratel (*Mellivora capensis*), jungle cat (*Felis chaus*), rusty spotted cat (*Prionailurus rubiginosus*), ruddy mongoose (*Herpestes smithi*), common Indian mongoose *(Herpestes edwardsi)* and small Indian civet (*Viverricula indica*). Major wild prey species were chital (*Axis axis*), sambar (*Rusa unicolor*), nilgai (*Boselaphus tragocamelus*), wild pig (*Sus scrofa*), chinkara (*Gazella bennettii*) and four horned antelope *(Tetracerus quadricornis)*. Chital constituted about 91% by number, and 78% by biomass, of the wild ungulate community in Gir [34].

We conducted this study within 725 km^2^ of the western part of the Gir PA, covering the entire tourism zone in the western part of the wildlife sanctuary and about 30% of the National Park between December 2014 to April 2015. Our study area encompassed livestock grazing areas of a cluster of nine *ness*es (pastoral hamlets) and four forest villages. Lion centric tourism is an important source of revenue for the Gir Protected Area and about 1.2 million tourists visit Gir annually [32]. Thus, the study area covered a wide range of values for all the potential covariates of lion density. Each *Maldhari* (local pastoralist communities) family rears about 33 (SE 3) livestock of the regionally famous indigenous breeds like Jafrabadi buffalo (*Bubalus bubalis*) and Gir cattle (*Bos indicus*) [30]. Livestock constituted between 14–25 (within the PA) to 40% (outside the PA) of lions’ diet through predation and scavenging [30,35]. Owing to religious sentiments most livestock are not consumed, and dead livestock are dumped outside *ness*es (*Maldhari* bomas) by *Maldhari*s. In order to minimize predator movements near the *ness*es and to maximize lion sightings within the tourism zone, park rangers, during the course of this study, collected livestock carcasses from ness sites and from villages along the close periphery of the Gir PA and moved them to specific locations within the tourism zone (referred to as baiting sites hereafter) to enhance lion sightings for tourists.

### Field methods

To systematically distribute sampling effort and avoid spatial “holes”, we divided the study site into sampling units (grid cells) of 25 km^2^ (n = 29), about half the size of an average home range of female lions (i.e., 48 km^2^, [36]). This eliminated any spatial holes in sampling and ensured that all lions within the study area had reasonable probability of being sampled (Fig 1). Each grid was visited on 10 different occasions and searched between December 2014 to March 2015. Lions were searched either on foot or by 4-wheel drive vehicle. During day time lions were known to restrict themselves within forested habitats which act as lion refuges [37], even within forests, as the day progresses and temperature increases, lions tend to use cool shady areas [36]. Professional trackers located lions using pugmarks, fresh scat, roars, prey alarm calls, crow/vulture assemblages on kills, and by visiting probable lion habitat patches. Searches were primarily conducted during early mornings and late evenings when lions were most active. The polygon search method is an extension of the SECR, wherein the detectors are “active” compared to passive detectors like stationary camera traps. Once encountered, lions (> 1 years of age) were approached to within 10–30 meters to get clear photographs of the vibrissae pattern and other permanent body marks for identification. Photographs were taken with a Canon HX 50 zoom camera [Canon India, Gurgaon, India] at 90° to the face from either side as well as a frontal view for individual identification based on vibrissae pattern, ear notches, and other permanent facial characteristics [19]. The vibrissae spots were allocated to specific predefined positions in a graphical representation, while notches on the ears were given specific positions like the dial of a clock (S1 Fig). Both, vibrissae spots and ear notch calibration, allow a quantitative evaluation [19]. For each lion sighting; time, date, geographic coordinates, gender, age class [23] and associated animals were recorded. These data along with photographs of each lion were then archived in Program Lion [38]. Program Lion allows to search the database to match lions that have high probability of being the same individual based on the above criteria. Based on matches of lions by the software, the closest matched lions were further verified from photographs of each lion sighting stored in the database and a final decision was made if the sightings were of the same individual or of different lions. Lions that were found together either sharing kills, socially interacting, allogrooming and otherwise being comfortable with each other and their cubs were considered to be of the same group [39]. The age class for lions [23] estimated at the time of first sighting was used throughout this field study duration of four months. A continuous track of the search path was recorded with a handheld GPS device (Garmin GPS Etrex 30; Garmin International, Kansas, USA). We computed cub to breeding age female ratio and proportion of breeding adult females in the population. Amongst lionesses in a pride, cubs are often cared for by related individuals and sometimes communally suckled. Therefore, ascertaining mothers of cubs was not always possible. However, this was unlikely to bias our ratios of cubs to breeding age females and breeding females in the population. Since sampling was done on multiple occasions and each lion sighted was individually identified and included only once for computation, we used sampling without replacement to compute the variance on these ratios [40].

Distance sampling [41] on foot line transects was used to collect data on herbivore spatial density. One to two transects were randomly oriented (but not facing east to avoid being blinded by the rising sun) in every sampling unit of 25 km^2^. Start and end point of all the transects along with their bearing were based on demarcating a line length of 2.5 to 4 km within the sampling grid. The start point was at a convenient accessible location to permit sampling during early morning hours. A total of 35 line transects were walked early morning with two to three people in each grid accounting for 101.5 km of walk effort. With every encounter of ungulates, we recorded the bearing of the animal(s) by using a see-through compass (Suunto KB 20), radial distance to the animal group centre using laser range finder (Bushnell RX 1000), and the geographical coordinates of the sighting with a handheld Global Positioning System (GPS) device (Garmin eTrex 30).

### Analytical methods

#### Spatially explicit lion density

We estimated lion density using maximum likelihood based spatially explicit capture-recapture (SECR) polygon search method [27,28]. SECR has the advantage over traditional CMR in that it uses information from spatially referenced ‘detectors’ [42], to model spatial density directly from the data [43]. The detection process is represented by a mathematical function that describes an animal's declining probability of being detected as we move further from its activity center. A simple detection function has the parameters i) lambda (λ_0_) which is the detection probability at the grid which contains the activity centre of the animal and ii) sigma (σ) which is the spatial scale of detection and decreases as distance increases from the activity centre [27]. In traditional CMR the density is calculated as $\hat{\text{D}}=\hat{\text{N}}/\text{ESA}$, where $\hat{\text{N}}$ is the estimated population size after correcting for detection and ESA is the effectively sampled area estimated using a buffer of half mean maximum distance moved by resighted individuals (½ MMDM) on the outermost lion locations [44]. While density. $\hat{\text{D}}$ is integral to the fitted models in SECR and calculated as a spatial point process based on the distribution of activity centres, fitted with a distance-based declining detection function [45].

The data in the polygon search method are organised as actual geographic location of animal detections, and the “trap file” constituted by the geographical coordinates of the polygon vertices representing square polygons sufficiently small to model the detection process [27]. In our case this trap file was constituted by vertices of 5 km^2^ square grids.

We arranged individual encounter histories using a standard SECR polygon detector matrix consisting of individual lion sighting locations and gender and group size as detection covariates [42], at a resolution of 5 km^2^. Adult male lions in the Gir PA occurred either solitarily or as coalitions of two or three, had home range sizes about four times larger than those of the females, and spent a major part of their time patrolling their territories, often scent marking and roaring while patrolling [46]. Lionesses occurred in larger groups with smaller ranges [36]. We hypothesized that detection parameters λ_0_ and σ would likely depend on gender based differential movements and group size and therefore modeled them as covariates in SECR. Juvenile and sub-adult male lions that lived in mixed groups with females were allocated the group size of the mixed group. We defined six *a priori* models (S1 Table) and evaluated their fit to our data (S2 Table) using maximum likelihood in package “secr” [47], a package developed in program R (R Core Team 2013) and selected the best fitted model based on AIC [48]. Subsequently, we estimated the abundance of lion by using argument “region.N” within the “secr” package [47] which provides the number within the spatial region of inference.

Since we conducted our search as 10 discrete occasions in time across each sampling grid, we were able to analyze our data using both SECR as well as traditional CMR. For traditional CMR, data were arranged in the traditional X matrix [17] with each individual lion marked as "1" when sighted in that occasion and as "0" when not detected in that occasion. Data were subsequently analyzed in MARK [49] under closed population assumption. We modeled capture probability of lions and an interactive term with gender and group size as covariates in Huggins closed capture models [50]. We ranked models using AIC [48]. Lion density was calculated by dividing the population size obtained from traditional CMR by the area of the 29 sampled grids and by the traditional ½ MMDM buffered polygon. The MMDM is computed from all lions that have been observed more than once, by considering the maximum displacement between two observations of the same lion. MMDM observations from all lions are then averaged and halved to compute ½ MMDM [44,51]. The ½ MMDM buffer was used to clip lion habitat surrounding the 5 km^2^ grids used for analysis to provide an estimate of effectively trapped area (S2 Fig).

#### Estimating prey density

We estimated chital and sambar density using conventional distance sampling (CDS) [38] in program DISTANCE [52] as well as density surface modelling (DSM) [29,53], in R using the package “dsm” [29]. For the DSM analysis all line transects were subdivided into segments of 400 m with their respective detections in each segment. A segment length of 400 m was considered ideal since it was of appropriate size to match the spatial resolution of eco-geographical covariates and sufficiently large to have reasonable number of segments with observations of ungulates within them so as to model detection. A detection function of half normal and hazard rate were fitted with combinations of hermite polynomial, cosine and simple polynomial to best explain animal detections with increasing distance along the line transect segments based on AIC and tests for goodness of fit of the models to the data [41]. Ungulates were likely to respond to terrain complexity, vegetation density [54], water availability [55] and human disturbance [56]. Sambar are known to utilise rugged and hilly terrain [57], whereas chital prefer flat valleys [58] and avoid anthropogenic disturbances [56]. Therefore, we use the following eco-geographical covariates to model spatial density of chital and sambar at a fine grid of 0.25 km^2^: (i) distance to water in meters, (ii) elevation above msl in meters, and (iii) Normalized Difference Vegetation Index (NDVI) to surrogate vegetation productivity (S3 Table). Following Hedley and Buckland [59], a count method was used for the spatial modelling process, wherein segment-specific counts of individuals were modelled as a function of the segment-specific eco-geographical covariates (S3 Table) with generalised additive modelling (GAM) [29,53]. To avoid over dispersion of the predictions, we used Tweedie distribution models [60]. Based on the visual comparison of residual fits, we specified the ϴ parameter as 1.2 [61]. Species-specific models were selected based on their AIC scores [48] and percentage deviance explained [29] (S5 Table). Spatial distribution maps of prey were generated based on the best fit model.

#### Modelling lion spatial density

Spatial lion density was a priori expected to be positively correlated with prey density [62] proximity to baiting sites, and productive flat valleys [63], while being negatively correlated with human use indices [4]. We used actual measures as well as surrogate indices of these parameters at the spatial scale of five km^2^ in Arc Map 10.2 (ESRI 2011) as follows: 1) density of major lion prey in grids, 2) distance of grids to baiting sites, 3) average elevation of grids in meters above msl, 4) distance to water sources in meters, 5) distance to night lights to surrogate proximity to human habitation (human footprint).

We used exploratory data analysis to evaluate relationships of these covariates with SECR lion density by inspecting scatterplots. Covariates that showed a relation with lion density were natural log transformed to conform to linearity and z-standardized [64]. Natural log transformed SECR lion density was subsequently modelled as a response to these natural log transformed variables using linear models (LM) [65] in base R (R Core Team 2013) and using the package “Rcmdr” [66]. We tested ten models based on hypothesises that explained lion density as a function of food resources (both natural prey and provisioned), habitat, and human impact. Model selection was based on AIC scores (Table 4).

## Results

### Lion demography

We obtained 368 detections of 67 individual lions (28 males and 39 females) belonging to 31 groups, with a sampling effort of 4,797 km of search within four months. A total of 15 cubs, 2 juvenile males, 3 sub adult females, 1 sub-adult male, 8 young adult females, 7 young adult males, 18 prime adult females, 10 old adult females, 11 prime adult males and 7 old adult males were encountered. In total, 31 groups were detected 163 times. The number of sightings per individual lion ranged from one to seven (average 3.04, SE 0.16) with an encounter rate of 0.076 (SE 0.01) lions per km searched. The average number of lions sighted on any one occasion (from a total of 10 occasions) was 36.8 (SE 5.61) lions. When plotted against cumulative lion sightings, the number of unique lions sighted showed an asymptotic curve, suggesting adequacy of sampling (S3 Fig). The ratio of cubs (< 1 year old) to breeding age lionesses was 0.41 (SE 0.05). The ratio of lionesses with cubs to adult lionesses without cubs was 0.36 (SE 0.05). Detection corrected sex ratio of female:male lions was 1.14 (SE 0.02). Lion groups ranged from one to six adults during our study, with a mean group size of 2.16 (SE 0.22) individuals/group, {2.52 (SE 0.36) for females (with juvenile and sub-adult males) and 1.71 (SE 0.15) for males}.

**Fig 2:**
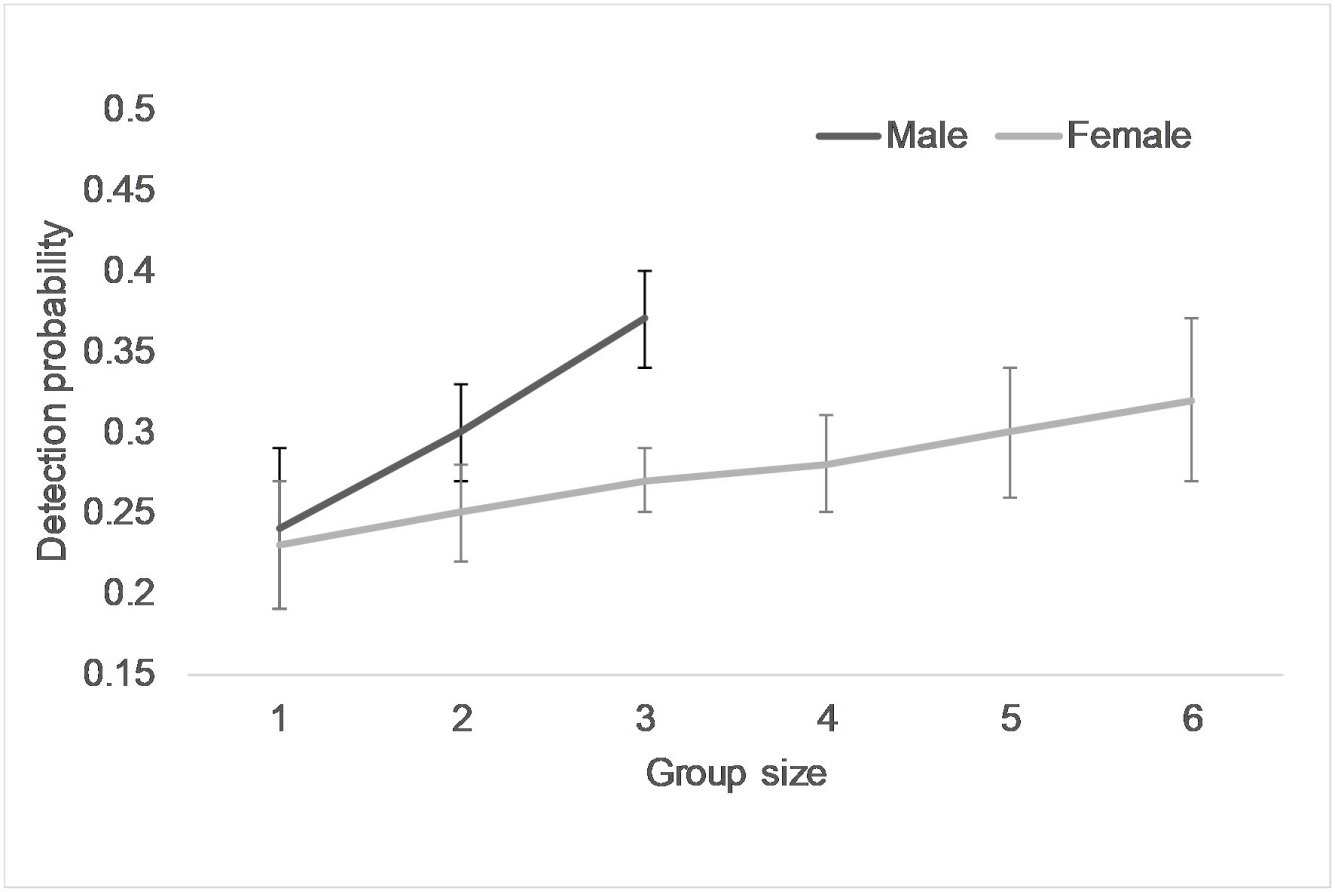
Average detection probability of male and female lions from different size groups estimated from closed population capture-mark-recapture in program MARK.

The SECR model space of 8 km buffer included 1081 km^2^ of lion habitat (S2 Fig). Lion (> 1 year) spatial density was estimated at 8.53 (SE 1.05)/100 km^2^ (Table 1) from the best model (λ_0_~sex, σ ~sex) (S2 Table). Density of male lions was lower than that of lionesses (Table 1). The probability of detecting males within grids containing their activity centres (λ_0_) during our study was certain while that for lionesses this detection probability was reasonably high (Table 1). The value of σ for male lions was double than that estimated for lionesses (Table 1). With traditional Huggins closed population CMR the best model included the effect of gender interacting with group size on capture probability (S4 Table). We found capture probability for males to differ from females and increase at a faster rate with increasing group size (Fig 2). The lion population in the study area was estimated at 70 (SE 3.60) with 41 (SE 1.54) females and 29 (SE 1.02) males by Huggins closed population. The area of the 29 sampled grids was 725 km^2^, while ½ MMDM buffer width was computed to be 4.5 (SE 0.68) km and the lion habitat effectively sampled within this ½ MMDM buffer was 747 (SE 26.56) km^2^. Therefore, CMR based lion density was computed to be 9.65 (SE 0.49) lions/100 km^2^ considering the area of the sampled grids and 9.37 (SE range 8.54 to 10.26) lions/100 km^2^ by using ½ MMDM approach.

**Table 1:** Parameters of lion density (lions per 100 km^2^) and abundance computed from individual lion sightings in a spatially explicit capture recapture (SECR), using polygon search maximum likelihood framework and traditional capture mark recapture using 10 sampling occasions.

Method	Abundance	Density	Gender	Gender specific density	λ_0_	σ (km)
SECR	67 (SE 8.19)	8.53 (SE 1.05)	M	3.07 (SE 0.58)	1 (SE 0.11)	5.32 (SE 0.33)
			F	5.45 (SE 0.87)	0.60 (SE 0.04)	2.55 (SE 0.12)
CMR	70 (SE 3.60)	9.37 (SE range 8.54 to 10.26)	M	4.14 (SE range 3.60 to 4.14)	-	-
			F	5.46 (SE range 5.07 to 5.89)	-	-

### Prey abundance

**Fig 3:**
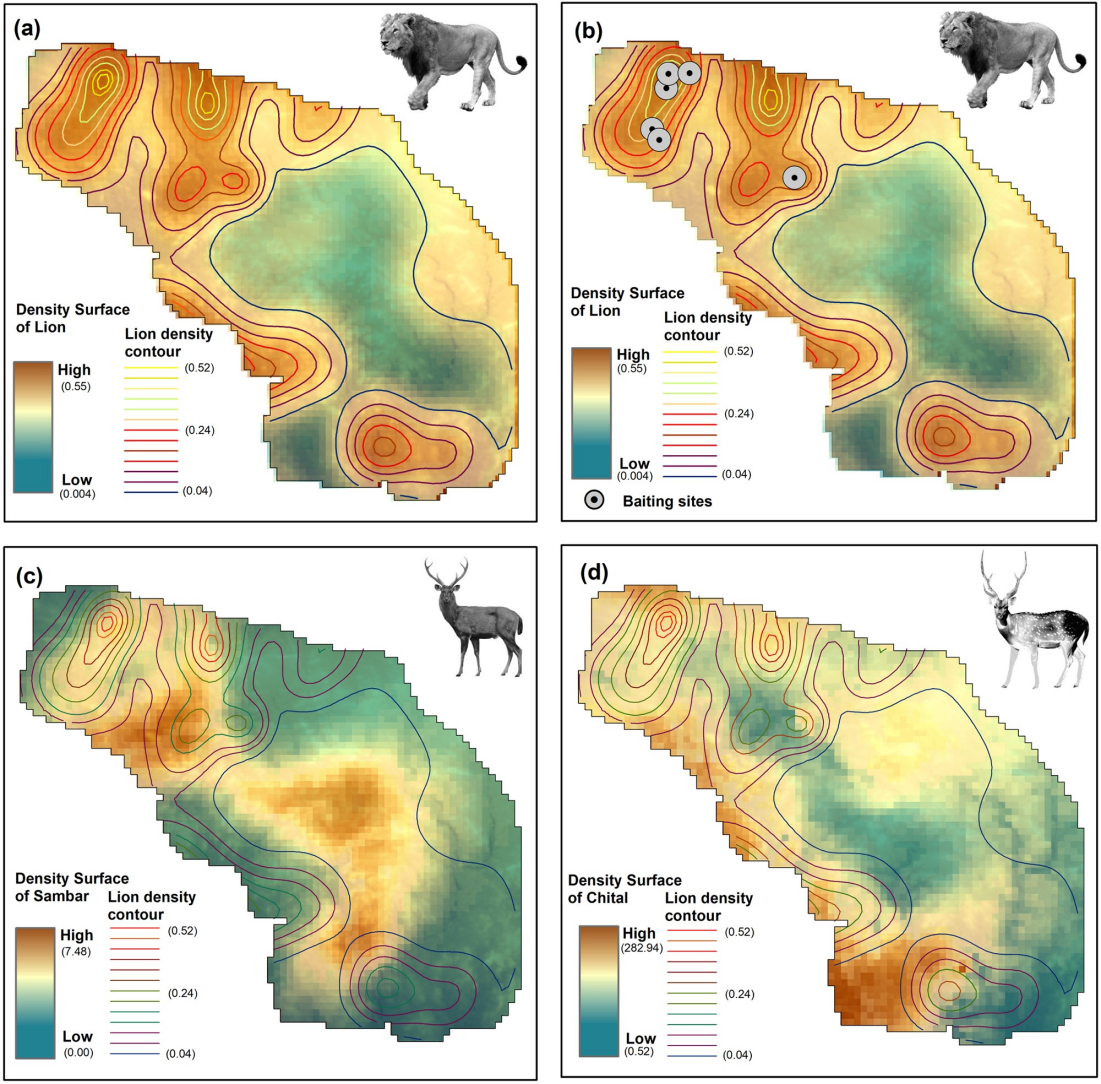
a) Spatially explicit density of Asiatic lions (8.53, SE 1.05 per 100 km^2^) depicted at 0.25 km^2^ grids where density ranged between 0.004 to 0.55 lions per km^2^; b) Density contours of Asiatic lion overlaid with baiting sites, c) Density surface of sambar depicted at 0.25 km^2^ grids where density ranged between 0 to 7.5 km^2^, overlaid with lion density contours and d) Density surface of chital at 0.25 km^2^ grids where density ranged between 0.53 to 283 km^2^ overlaid with lion density contours in western Gir Protected Area, India.

We obtained 98 sightings of chital groups and 25 sightings of sambar groups. Hazard rate models with polynomial adjustment functions best explained the observed detection data of both chital and sambar. The Chi square (χ^2^) goodness of fit test suggests that the data fit the model well with χ^2^ = 0.16, P = 0.98 for chital and for sambar the values were χ^2^ = 0.038, P = 0.98. Estimated chital density was 63.29 (SE 10.14) km^2^ and sambar density was 3.84 (SE 1.07) km^2^ (Table 2). The density estimated through DSM was similar to that obtained by CDS with chital at 58.75 (SE 22.17) km^2^ and sambar at 4.73 (SE 1.48) km^2^. Spatial variation in chital density was best explained by the additive effects of NDVI and proximity to water, whereas density of sambar was explained by elevation (Table 3, Fig 3, S5 Table, S4 Fig).

**Table 2:** Density (per km^2^) of major prey species of lion estimated by using conventional distance sampling (CDS) in program distance.

Species	Detection function	Density in km^2^	DS	Average cluster size	Detection probability	Encounter rate	ESW	χ^2^ *p*
Chital	Hazard rate polynomial	63.29 (SE 10.14)	7.58 (SE 0.93)	8.34 (SE 0.85)	0.73	0.96	63.65 (SE 3.64)	0.98
Sambar	Hazard rate polynomial	3.84 (SE 1.07)	2.18 (SE 0.56)	1.76 (SE 0.19)	0.71	0.24	56.40 (SE 8.44)	0.98

DS—Estimated density of groups, ESW—Effective sampling width, χ^2^ ***p***—Chi square goodness of fit *p* value.

**Table 3:** Parameters of the best model along with their effective degrees of freedom (edf) and statistical significance that explained the spatial densities of lion prey (chital and sambar) in western Gir Protected Area.

Species	Detection function	Parameters	edf	p-value
**Chital**	Hazard-rate polynomial	x,y	26.20	0.002
		NDVI	0.56	0.170
		water	10.26	0.002
**Sambar**	Hazard-rate polynomial	x,y	2.67x10^-5^	0.76
		elevation	1.73	0.07

NDVI—normalized difference in vegetation index surrogating vegetation productivity, elevation—elevation, edf—effective degrees of freedom

### Spatial covariates of lion density

Simple linear regression and a scatterplot of lion density showed relationships with elevation, distance to baiting sites and proximity to human habitation. Chital spatial density was marginally significant (P = 0.06) with a very weak relation with lion density, while there was no relationship with spatial density of sambar, distance to water sources, or vegetation density (S5 Fig). The model with additive effect of “distance to baiting sites”, “elevation” and “proximity to human habitation” was found to best explain variation in lion density. The linear model fit our data well with no patterns observed in the residuals and having a coefficient of determination R^2^ = 0.43 (Table 4, S6 Fig). Lion density declined with increasing distance to “baiting sites” {-0.53 (SE 0.07), p = <0.001}, lions were observed to use lower elevation valley habitats more often {elevation, -0.28 (SE 0.08), p = <0.001} and lion density was high closer to human habitations {distance to human habitation, -0.25 (SE 0.10), p = 0.01} (Table 4).

**Table 4:** Covariates that explained spatial variation in lion density within western Gir Protected Area.

Model No	Model covariates	*β* Estimates	*p* value	Adj. R^2^	AIC	ΔAIC
1	(Intercept)	-0.0 (SE 0.06)	1	0.43	374.99	0
	Bait (β1)	-0.53 (SE 0.07)	<0.001			
	Human habitation (β2)	-0.25 (SE 0.10)	0.01			
	Elevation (β3)	-0.28 (SE 0.08)	<0.001			
2	Bait + Elevation			0.41	379.35	4.36
3	Bait + Human habitation			0.4	384.52	9.53
4	Bait + Chital + Sambar			0.36	393.84	18.85
5	Bait			0.25	417.87	42.88
6	Human habitation			0.22	425.58	50.59
7	Prey density			0.05	459.81	84.82
8	Chital			0.021	462.22	87.23
9	Sambar			0.016	463.09	88.10
10	Water			0.007	463.5	88.51

Bait—Ln (distance to baiting sites in m); Human habitation—Ln (distance to human habitation in m); Elevation—Ln (elevation in m), Prey density—Ln (chital per km^2^) + Ln (Sambar per km^2^), Water—Ln (Distance to Water in m)

## Discussion

We demonstrate a robust approach to assessing the density of Asiatic lions by first identifying individual lions reliably using replicable criteria [18,19] and subsequently estimating density through polygon search method in a maximum likelihood based SECR framework [24]. We used photographic records to uniquely identify each lion through their vibrissae pattern and permanent body marks using program "Lion" [38] that allowed data archiving, retrieval and easy algorithm-based digital comparisons. This approach permits rapid comparisons of capture histories of individual lions and is useful for CMR, SECR, demographic and behavioral studies. Detection of larger groups was greater than smaller groups (Fig 2). We expected male and female lions to have different detections due to behavioral differences like higher frequency of vocalization, as well as movement parameters due to larger ranging and patrolling for territory defense among males and differential resource requirements between the genders. In our study there were only three groups with dependent males (i) three females with two juvenile males, (ii) five females with one juvenile male and (iii) two females with one juvenile male. The small number of such mixed groups with a small number of young males did not confound our results. Besides young males prior to dispersal tend to be skittish and elusive and have detections similar to that of females. Therefore, after considering these mixed groups as “female” groups our results were in agreement with our hypothesis with adult males being easier to detect and having a larger σ compared to females (Table 1).

The SECR approach is relatively new compared to traditional CMR. Both approaches should provide similar unbiased estimates of abundance and in our case, even density estimates were not significantly different. However, search encounter methods are often conducted in an unstructured manner [67–69] and usually cannot be analysed by traditional CMR approaches. We, therefore, adopted a robust sampling design by spatio-temporal replication of the study area through multiple visits to the same spatial grid (10 sampling occasions) in a systematic temporal survey design amenable to analysis using traditional closed population capture-mark-recapture models [70] as well as by SECR. By a good experimental sampling design and by accounting for known sources of variation likely to affect lion detections which included gender, and group size in CMR and SECR we obtained similar estimates of abundance by both approaches. This lends further support to the robustness of our approach for estimating lion abundance.

It is often debated whether the density surface generated by SECR actually reflects the spatial density of the target species when modelled without appropriate covariates or systematic sampling [71]. However, in our case, since the observational process involved detection of greater than 96% of the entire lion population using a systematic sampling design and modelled at a relevant spatial scale of 5 km^2^, the resulting SECR density surface would not vary from the actual density surface by any significant extent.

The two major natural prey species of Asiatic lions were found to have a distinct spatial distribution and density in the landscape. Habitat heterogeneity of the landscape primarily explained distribution of chital and sambar with the former preferring valley habitats with good vegetation and water availability [57] while the latter preferring rugged and elevated areas [72].

Earlier studies on lion diet had shown that lions subsisted primarily on natural prey in the protected areas [30,35]. We therefore, expected lion density to be determined by natural prey distribution. Contrary to our expectation, lion spatial density was poorly correlated to the density and distribution of its principal prey species (Table 4, Fig 3). This was likely since lions in the tourism zone got assured food through provisioning and natural prey probably did not regulate lion movement or density.

Keith’s equation [73], (*W* = *U*(*λu* − 1)/*K*), where, W = number of predator, U = number of ungulates, λu = finite rate of population increase for an ungulate, K = annual ungulate kill per carnivore, provides a good rationale for computing carnivore numbers based on kill rates and the prey’s finite rate of population increase so as to maintain an equilibrium. We considered a predation rate of one chital size prey per lion every 3 days [30] and a lambda value of 1.3 for chital [74], then a population of about ~400 individuals of chital size prey are required for each lion per year, if lions were to subsist entirely on chital without causing declines in the chital population. In western Gir, the ratio was 600 to 700 chital per lion. Gir also has a good density of leopards, which subsist on the same prey as lions [75]. Considering both lion and leopard densities and the current trend of increasing livestock in the lion’s diet, the large carnivore population in Gir were unlikely to cause prey depletion. This predator-prey ratio shows that provisioning was not essential for maintaining the lion population. Since wild prey were available in good numbers across the landscape, they seemed to have little influence on lion density at the fine spatial scale. Lion density concentrations were mostly determined by assured food provisioning at baiting sites at the local scale. Distance to baiting sites had the largest magnitude of influence compared to other factors like elevation and proximity to human habitation. The practice of dumping livestock carcasses from *nesses* and forest villages at tourist viewing spots in an attempt to mitigate lion-human conflict and increase lion sightings to tourists, resulted in larger prides that resided in close proximity to such areas. Provisioned prides were larger ranging from 5–7 (adult females) compared to the average adult female group size reported from the same areas earlier at 1.52 (SE 0.07) [76]. Such changes in local density and ranging behavior have also been reported in provisioned black bears in Quebec, Canada [77]. Practices of human food provisioning is known to have significant influence on behavioral [78,79] and functional response [77] of large carnivores. The artificially localized high density of lions could also have impacts on their social dynamics as well as on local prey populations. In many prides that were provisioned younger lions were observed to lack the predatory skills required to hunt, as cubs were fed with dumped carcasses with regularity and grew up as scavengers. Such animals that lack skills to hunt often come in conflict with humans, as after they disperse from the tourism zone and are no longer provisioned they try to kill livestock (easier prey) and can also become a danger to human lives [77,80].

Though lions do occur in rugged terrain they were observed to prefer plains [63]. Our study showed a similar preference by lions in Gir which had a positive relation with flat valley habitats. The positive relation with human footprint index is an artifact of how the index is computed and its values within the lion habitat (the modeled space for the relationship). Lions like most large carnivores, have soft padded feet and prefer to walk on smooth surfaces [81]. Village roads, cart tracks that contribute to the human footprint index are also used by lions for patrolling, and commuting, and visiting Ness sites in search for scavenging opportunities on dumped livestock carcasses, resulting in a relationship between lion density and human footprint index.

Various techniques have been used and can potentially be used to estimate the abundance of lions. These include: 1) Track counts [82,83] and playback surveys [84,85], which serve as indices of abundance and can be used to monitor population trends under very restrictive assumptions [86] and standardized designs. However, they have the potential to be calibrated against absolute abundance for quick and economic landscape scale surveys [87]. 2) total counts [16,39,88] which are currently the officially approach for evaluating the status of Asiatic lions are the least reliable at landscape scales due to the methods’ inherent inability of addressing detection probability as well as double counts. The currently used total counts does not use individual identification of lions. 3) Traditional mark-recapture based on individual identifications of lions [19,89–91]. This approach is superior to total counts as it gives a measure of precision by addressing detection probability. Multiple observations on same individuals (re-captures) are used in a statistical framework to estimate detection probability, that is subsequently used to account for lions that may not be sampled in total counts. Traditional CMR method has been successfully demonstrated for both Asiatic [19,22] and African lions [90,91]. 4) Transect based distance sampling [92, 93] and camera trap based distance sampling [94], have the potential of being used for estimating lion density. However, distance sampling requires large number of encounters for fitting detection functions and are generally used for ungulates (that are more abundant) rather than carnivores (that have fewer encounters). 5) Faecal DNA based genotyping has the potential for individual identification of lions [95]. This approach if used in an SECR framework can provide reliable spatial estimates of density (much like the one presented in this paper). However, Asiatic lions are believed to be highly inbred [96] and a microsatellite panel that can potentially have the power to differentiate between closely related Asiatic lions has as yet to be developed and tested. Besides, currently the errors associated with faecal DNA based genotyping are high [97,98]. Elliot and Gopalaswamy (2016) [69], estimated lion densities in the Maasai Mara National Reserve, Kenya, using opportunistic lion sightings in a polygon search SECR and demonstrated the potential and usefulness of the approach. The work in Maasai Mara was done contemporaneously with this work in Gir. By incorporating an additional vibrissae row (row “C”) and calibrated ear notches for automated individual identification of lions using program “Lion” [38] along with an experimental design for sampling that was conducive for traditional CMR as well as SECR analysis, and by explaining lion density as a function of resources, we take this approach a step further. We propose that polygon search SECR can be used in place of total counts of lions as well as traditional CMR, for assessing their status within the entire Gir landscape. Such an approach would not only provide an estimate of abundance, but due to its spatially explicit nature, will be useful for site specific management and policy formulation. Our research was rather intensive as we visited each spatial sampling unit (25 km^2^) on multiple occasions for collecting data conducive for traditional CMR analysis as well, this is not essential for SECR. The SECR approach to estimating lion density across the Saurashtra landscape can be achieved by fewer visits using a stratified sampling approach making it a cost effective and efficient approach to evaluating the status of Asiatic lions. Since SECR is dependent on spatial recaptures and not on occasions of sampling visits [99]. Searching for lions requires the maximum effort for this exercise. Lions once located can be followed (relocated) with much smaller effort [30]. Several spatial locations (that are spatially independent after testing for autocorrelation) could be obtained by relocating/following lions, in place of temporal replicates required for traditional CMR. The use of inappropriate methods for assessing the status and trends of endangered species can have dire consequences [9]. A major issue with total counts is that they are not accompanied by an estimate of any error, as they don’t account for double counts and detection probability (which is assumed to be 1) and are dependent on the amount of effort invested. As there is no estimate of any error, there is no measure of bias or precision [100]. It would not be prudent to use such estimates for making conservation assessments and management decisions for endangered species [101] especially when scientifically robust approaches are available and demonstrated.

## Supporting information

S1 Table Different models tested in SECR for estimating lion density in western Gir Protected Area. (DOCX)

S2 Table Model selection statistics and SECR density estimates of lions (individuals per 100 km^2^) in the western Gir Protected Area. (DOCX)

S3 Table Details of spatial and attribute covariates used for assessing spatial density of Asiatic lions and their principal prey species in the western Gir Protected Area. (DOCX)

S4 Table Model selection statistics for abundance estimation of Asiatic lions in western Gir Protected Area, using Huggins’ closed capture models in a conventional mark-capture-recapture framework. Sex and group size were used as covariates to model capture and recapture probability.
(DOCX)

S5 Table Model selection and density estimates (number/km^2^) of major prey species (chital and sambar) in western Gir Protected Area, as estimated by density surface modeling. Combinations of different eco-geographical covariates were used to model spatial variation in abundance. Best fit models were selected based on their generalized cross validation scores (CV) and percentage of deviance explained.
(DOCX)

S1 Fig Identification of individual lions based on vibrissae pattern and other permanent body mark. (A) A depiction of vibrissae-based identification of individuals with other permanent marks. (B) A screenshot of the program “Lion”.
(DOCX)

S2 Fig Lion habitats within the ½ MMDM buffer to estimate density of lions in CMR overlaid with 25 km^2^ sampling grids. (DOCX)

S3 Fig Cumulative lion sighting plotted against sampling occasion to check adequacy of sampling. The cumulative sighting of both male and females is projected separately in the graph.
(DOCX)

S4 Fig Exploratory data analysis to evaluate relationships of covariates with SECR lion density by inspecting scatterplots. (DOCX)

S5 Fig Residual plots and qq plots for testing normality of the residuals. (DOCX)

S6 Fig Residual plots and qq plots for testing normality of the residuals of the best fit model explaining lion density as a function of distance to baiting sites, elevation, and distance to human habitation. (DOCX)
