# Supplementary material for: Spatially explicit density and its determinants for Asiatic lions in the Gir forests
Source: PLoS One. 2020 Feb 19;15(2):e0228374. doi: 10.1371/journal.pone.0228374 (PMC7029878; doi:10.1371/journal.pone.0228374)
Supplement: S1 Table — (DOCX) [file pone.0228374.s001.docx]

**Table S1. Different models tested in SECR for estimating lion density in western Gir Protected Area.**

| **Model No** | **lambda (λ_0)_** | **sigma (σ)** | **Description** |
| --- | --- | --- | --- |
| 1 | *Group size* | *Gender* | Detection probability at the grid with activity centre (λ_0_) as a function of group size, whereas scale parameter (σ) as a function of gender |
| 2 | *Group size* | *Constant* | λ_0_ as a function of group size with σ constant |
| 3 | *Gender* | *Gender* | Both λ_0_ and σ are functions of lion gender |
| 4 | *Constant* | *Group size* | λ_0_ as constant whereas σ as a function of group size |
| 5 | *Gender* | *Constant* | λ_0_ as a function of gender, whereas σ as constant |
| 6 | *Constant* | *Constant* | Null model, both the λ_0_ and σ are constant |
