# Supplementary material for: Spatially explicit density and its determinants for Asiatic lions in the Gir forests
Source: PLoS One. 2020 Feb 19;15(2):e0228374. doi: 10.1371/journal.pone.0228374 (PMC7029878; doi:10.1371/journal.pone.0228374)
Supplement: S2 Table — (DOCX) [file pone.0228374.s002.docx]

**Table S2.** Model selection statistics and SECR density estimates of lions (individuals per 100 km^2^) in the western Gir Protected Area.

| **Model** | **Lion Density** | **Gender** | **Gender Specific Density** | **λ_0_** | **σ (km)** | **Log likelihood** | **N par** | **AIC** | **ΔAIC** |
| --- | --- | --- | --- | --- | --- | --- | --- | --- | --- |
| λ_0_(sex), σ (sex) | 8.53 (SE 1.05) | M | 3.07 (SE 0.58) | 1 (SE 0.11) | 5.32 (SE 0.33) | -7719.19 | 5 | 15448.39 | 0 |
|  |  | F | 5.45 (SE 0.87) | 0.60 (SE 0.04) | 2.55 (SE 0.12) |  |  |  |  |
| λ_0_ (grp), σ (sex) | 8.59 (SE 1.05) | M | 3.35 (SE 0.63) | 0.69 (SE 0.07) to 0.80 (SE 0.09) | 4.84 (SE 2.48) | -7728.68 | 5 | 15467.37 | 18.98 |
|  |  | F | 5.24 (Se 0.84) | 0.69 (SE 0.07) to 0.80 (SE 0.09) | 2.67 (SE 1.43) |  |  |  |  |
| λ_0_ (.), σ (grp) | 8.21 (SE 1.01) | - | - | 0.82 (SE 0.05) | 3.22 (SE 1.57) | -7745.01 | 4 | 15498.03 | 49.64 |
| λ_0_ (grp), σ (.) | 8.15 (SE 1.00) | - | - | 1.00 (SE 0.12) to 0.70 (SE 0.07) | 4.03 (SE 1.59) | -7759.3 | 4 | 15526.63 | 78.24 |
| λ_0_ (.), σ (.) | 8.15 (SE 1.00) | - | - | 0.83 (SE 0.05) | 4.02 (SE 1.59) | -7761.83 | 3 | 15529.67 | 81.28 |
| λ­_0_ (sex), σ (.) | 8.17 (SE 1.00) | M | 3.38 (SE 0.64) | 0.87 (SE 0.07) | 4.01 (SE 1.58) | -7761.54 | 4 | 15531.1 | 82.71 |
|  |  | F | 4.78 (SE 0.77) | 0.79 (SE 0.07) | 4.01 (SE 1.58) |  |  |  |  |

grp- group size; sex – gender of the individuals male or female; λ_0_ – detection probability within the grid containing the activity center; σ – spatial scale parameter (km); N par – Number of model parameters; AIC – Akaike Information Criterion. When λ_0_ was modeled with group size, each group size resulted in a value of λ_0_ with SE. Here we present the smallest and largest values of λ_0_ in the models.
