# Supplementary material for: Spatially explicit density and its determinants for Asiatic lions in the Gir forests
Source: PLoS One. 2020 Feb 19;15(2):e0228374. doi: 10.1371/journal.pone.0228374 (PMC7029878; doi:10.1371/journal.pone.0228374)
Supplement: S3 Table — (DOCX) [file pone.0228374.s003.docx]

**Table S3.** Details of spatial and attribute covariates used for assessing spatial density of Asiatic lions and their principal prey species in the western Gir Protected Area.

| **Dataset** | **Description** |
| --- | --- |
|  |  |
| Normalized Difference Vegetation Index (NDVI) | 250m^2^ Advanced Very High-Resolution Radiometer (AVHRR) data, acquired from the National Aeronautics and Space Administration’s (NASA) Television Infrared Observation Satellite (TIROS) (http://science.nasa.gov/missions/ tiros/; accessed 03 March 2015). NDVI value of each pixel was used for the models. |
| Elevation | 90-meter resolution Digital Elevation Model (DEM) data with 2 bands generated by the Shuttle Radar Topography Mission [NASA, The National Geospatial-Intelligence Agency, and the German and Italian Space Agencies] (Rodriguez *et al.*, 2005). Elevation of each pixel in meters above mean sea level was used for models. |
| Water | Availability of natural and managed water holes. Ground validated and digitized data from the management of Gir Protected Area. Distance of each pixel in meters from a water source was computed and used in the model. |
| Night light | United States Air Force Defense Meteorological Satellite Program (DMSP) and National Oceanic and Atmospheric Administration’s (NOAA) Operational Linescan System (OLS) with a pixel size of 1 km^2^. (http://www.ngdc.noaa.gov/dmsp/sensors/ols.html; accessed 30 May 2015) (Elvidge *et al*., 1997). Distance of each pixel in meters from the night light source was computed and used in the model. |
