# Supplementary material for: Spatially explicit density and its determinants for Asiatic lions in the Gir forests
Source: PLoS One. 2020 Feb 19;15(2):e0228374. doi: 10.1371/journal.pone.0228374 (PMC7029878; doi:10.1371/journal.pone.0228374)
Supplement: S4 Table — Sex and group size were used as covariates to model capture and recapture probability. (DOCX) [file pone.0228374.s004.docx]

**Table S4.** Model selection statistics for abundance estimation of Asiatic lions in western Gir Protected Area, using Huggins’ closed capture models in a conventional mark-capture-recapture framework. Sex and group size were used as covariates to model capture and recapture probability. grp- group size; sex – gender of the individuals male or female

| **Models** | **AICc** | **ΔAICc** | **AICc Weight** | **Model likelihood** | **No. Par** | **Deviance** |
| --- | --- | --- | --- | --- | --- | --- |
| p=c ~ a (sex) + b(grp*sex) | 816.51 | 0.00 | 0.06 | 1.00 | 4 | 808.45 |
| p=c ~ a(sex) + b(grp) | 816.97 | 0.46 | 0.05 | 0.80 | 3 | 810.94 |
| p ~ a(sex) + b(grp)  c ~ a(sex) + b(grp) | 817.13 | 0.62 | 0.04 | 0.74 | 5 | 807.04 |
| p ~ a(sex) + b(grp*sex)  c ~ a(sex) + b(grp*sex) | 817.16 | 0.65 | 0.04 | 0.72 | 8 | 800.94 |
| p = c ~ a (.) + b(grp) | 819.71 | 3.19 | 0.01 | 0.20 | 2 | 815.69 |
| p ~ sex, c ~ sex | 820.79 | 4.28 | 0.01 | 0.12 | 4 | 812.73 |
| p = c (.) | 821.74 | 5.22 | 0.004 | 0.07 | 1 | 819.73 |
| p = c ~ sex | 821.80 | 5.28 | 0.00 | 0.07 | 2 | 817.78 |
