# Supplementary material for: Spatially explicit density and its determinants for Asiatic lions in the Gir forests
Source: PLoS One. 2020 Feb 19;15(2):e0228374. doi: 10.1371/journal.pone.0228374 (PMC7029878; doi:10.1371/journal.pone.0228374)
Supplement: S5 Table — Combinations of different eco-geographical covariates were used to model spatial variation in abundance. Best fit models were selected based on their generalized cross validation scores (CV) and percentage of deviance explained. (DOCX) [file pone.0228374.s005.docx]

**Table S5.** Model selection and density estimates (number/km^2^) of major prey species (chital and sambar) in western Gir Protected Area, as estimated by density surface modeling. Combinations of different eco-geographical covariates were used to model spatial variation in abundance. Best fit models were selected based on AIC scores and percentage of deviance explained.

| **Model** | **Detection function** | **Density** | **SE** | **AIC** | **Deviance explained** |
| --- | --- | --- | --- | --- | --- |
| **Chital DSM estimates:** |  |  |  |  |  |
| NDVI+Water | Hazard rate | 58.75 | 22.17 | 831.10 | 47.90% |
| Water | Hazard rate | 58.04 | 19.83 | 832.95 | 45.00% |
| NDVI+water+elevation | Hazard rate | 58.04 | 19.26 | 837.33 | 44.90% |
| NDVI | Hazard rate | 66.75 | 28.28 | 851.80 | 38.60% |
| Elevation | Hazard rate | 60.12 | 20.81 | 860.71 | 34.00% |
| Null | Hazard rate | 64.31 | 18.17 | 870.57 | 26.70% |
| **Sambar DSM estimates:** |  |  |  |  |  |
| Elevation | Hazard rate | 4.73 | 1.48 | 298.59 | 4.86% |
| Elevation+Water | Hazard rate | 4.47 | 1.41 | 314.63 | 3.74% |
| Water | Hazard rate | 4.07 | 1.35 | 332.61 | 0.00% |
| NDVI | Hazard rate | 4.07 | 1.35 | 332.61 | 0.00% |
| Null | Hazard rate | 4.07 | 1.93 | 378.88 | 0.00% |
