# Supplementary material for: Spatially explicit density and its determinants for Asiatic lions in the Gir forests
Source: PLoS One. 2020 Feb 19;15(2):e0228374. doi: 10.1371/journal.pone.0228374 (PMC7029878; doi:10.1371/journal.pone.0228374)
Supplement: S1 Fig — (A) A depiction of vibrissae-based identification of individuals with other permanent marks. (B) A screenshot of the program “Lion”. (DOCX) [file pone.0228374.s006.docx]

**Fig S1:** Identification of individual lions based on vibrissae pattern and other permanent body mark. (A) A depiction of vibrissae-based identification of individuals with other permanent marks. (B) A screenshot of the program “Lion”.

**
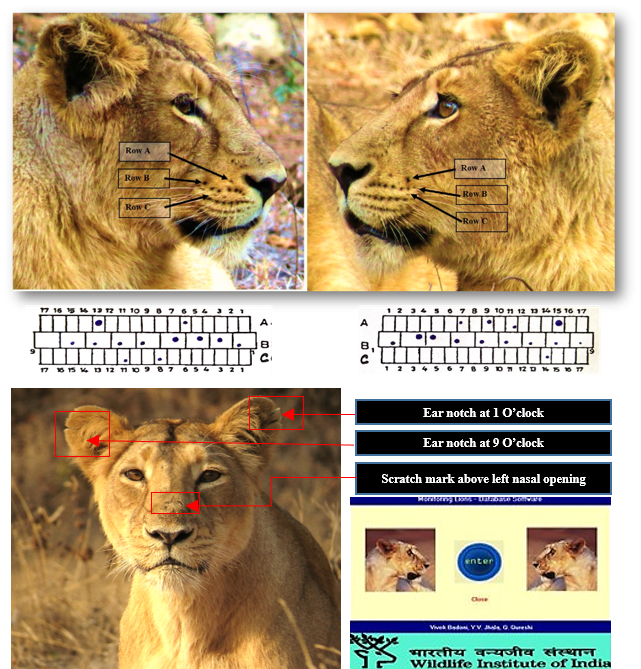
**

(A)

(B)

**
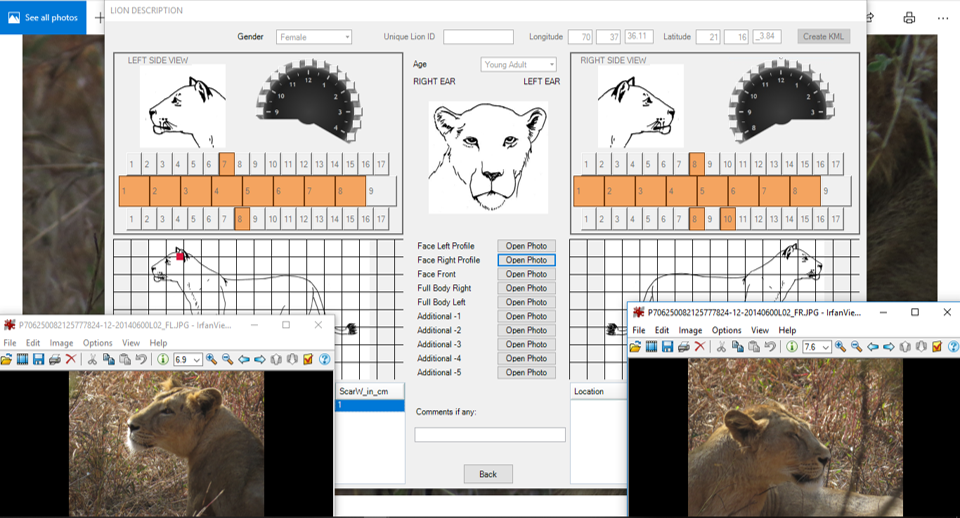
**
