# Supplementary material for: Spatially explicit density and its determinants for Asiatic lions in the Gir forests
Source: PLoS One. 2020 Feb 19;15(2):e0228374. doi: 10.1371/journal.pone.0228374 (PMC7029878; doi:10.1371/journal.pone.0228374)
Supplement: S3 Fig — The cumulative sighting of both male and females is projected separately in the graph. (DOCX) [file pone.0228374.s008.docx]

**Fig S3:** Cumulative number of lions recorded plotted against sampling occasion to check adequacy of sampling. The cumulative sighting of both male and females is projected separately in the graph.


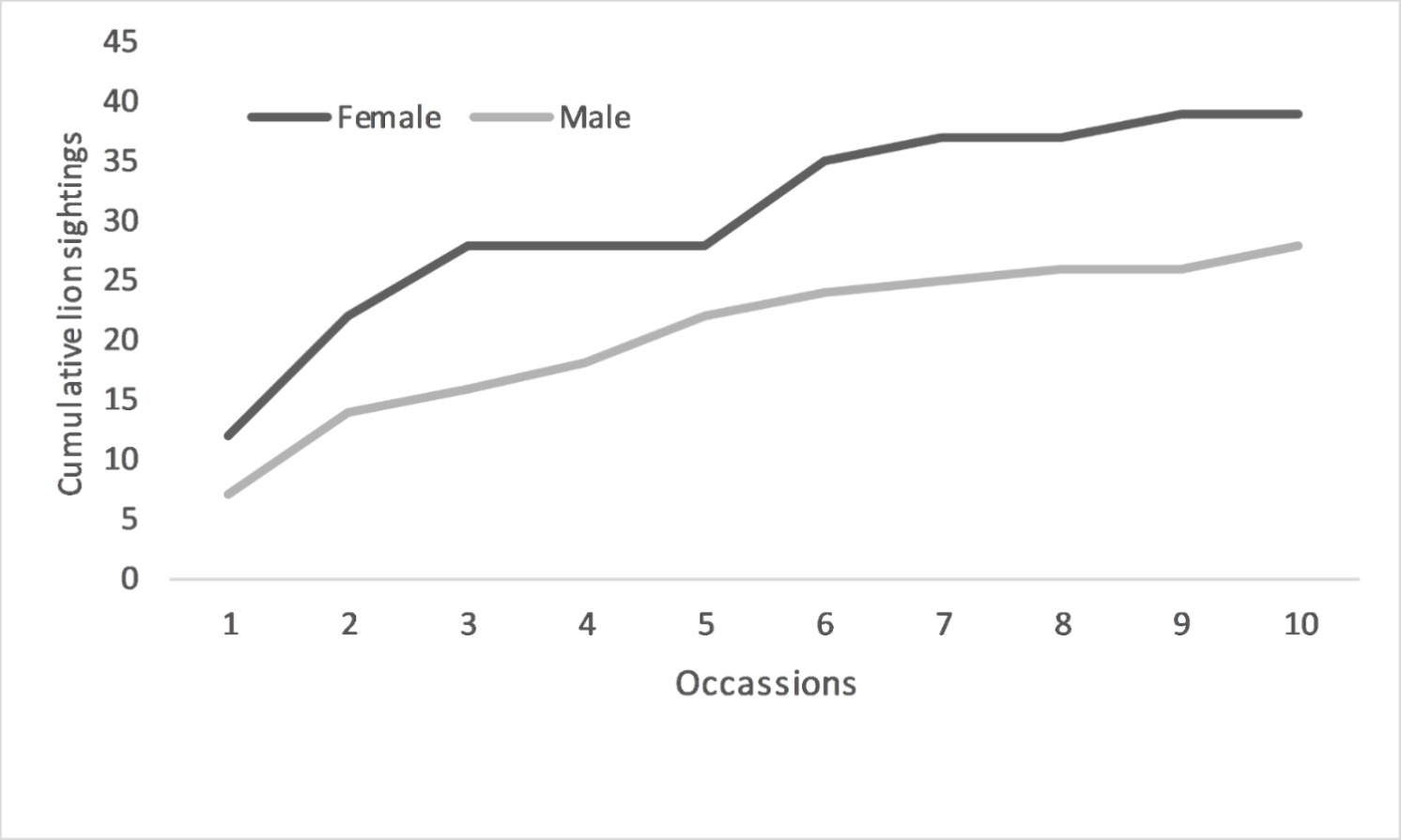


Cumulative number of lions recorded
