# Supplementary material for: Spatially explicit density and its determinants for Asiatic lions in the Gir forests
Source: PLoS One. 2020 Feb 19;15(2):e0228374. doi: 10.1371/journal.pone.0228374 (PMC7029878; doi:10.1371/journal.pone.0228374)
Supplement: S4 Fig — (DOCX) [file pone.0228374.s009.docx]

**Fig S4:** The graphs represent the partial effects of the significant predictors of the best model for (a) Chital and (b) Sambar using Generalized Additive Modeling with 95% confidence interval (grey shading). The ticks on the x axis represents the range of values for the variables in the surveyed area and the value within the parentheses on the y axis represents the effective degrees of freedom for each term.

a) **Chital**


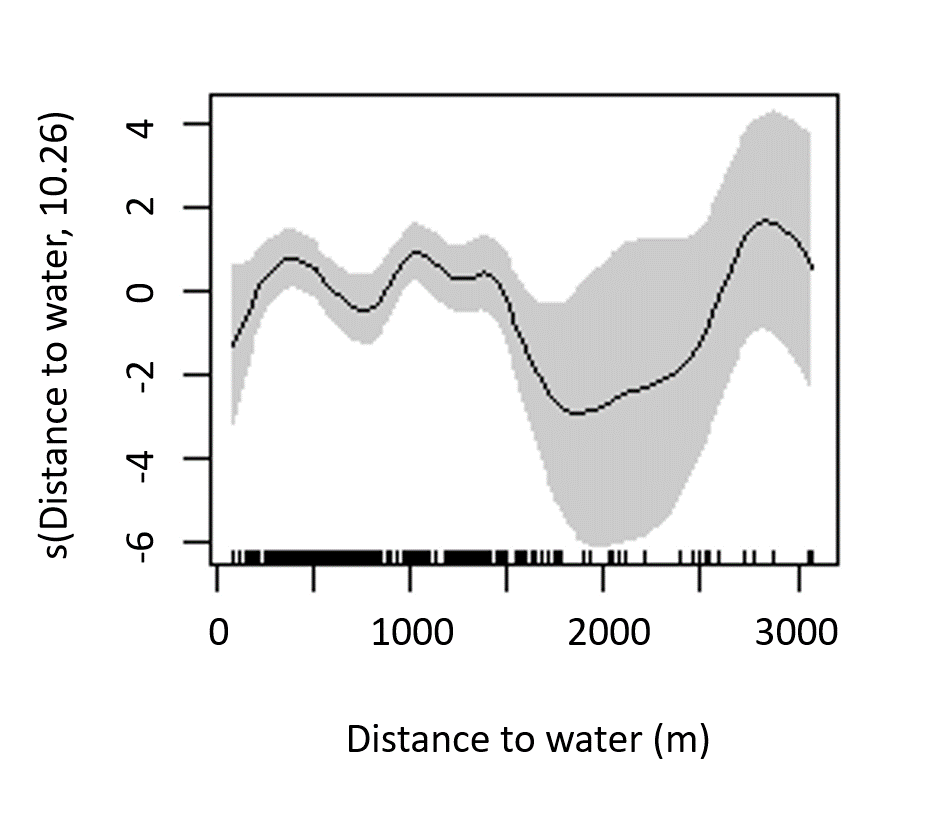


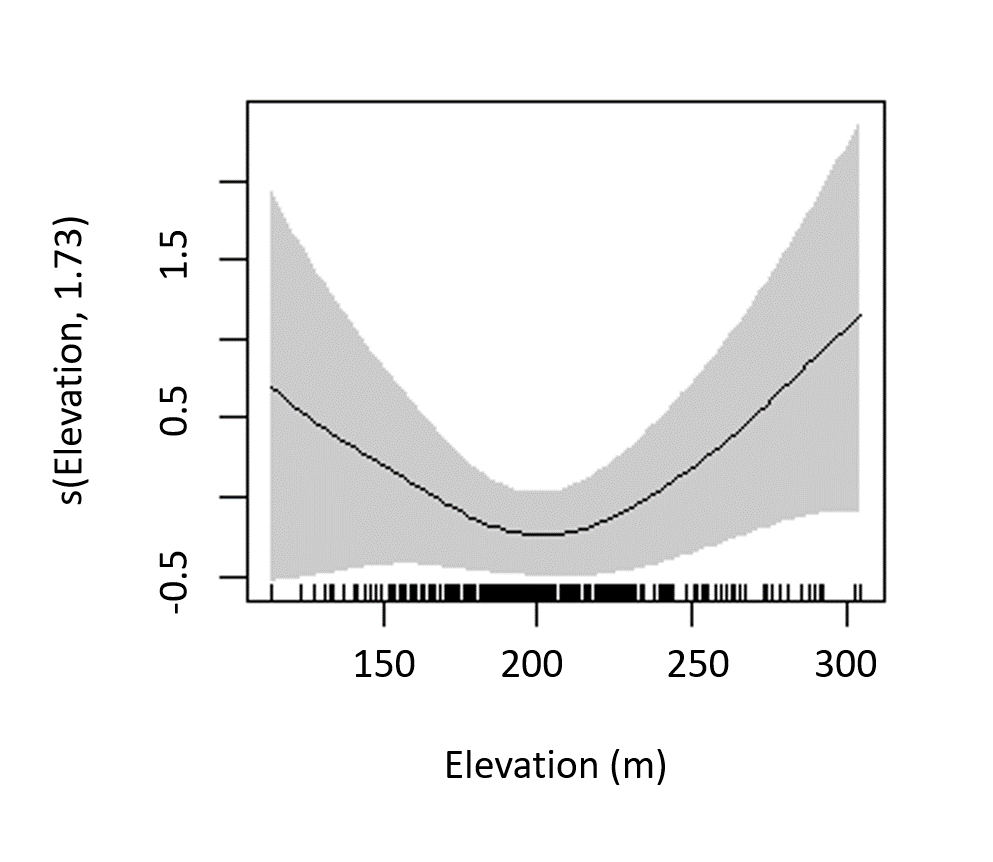

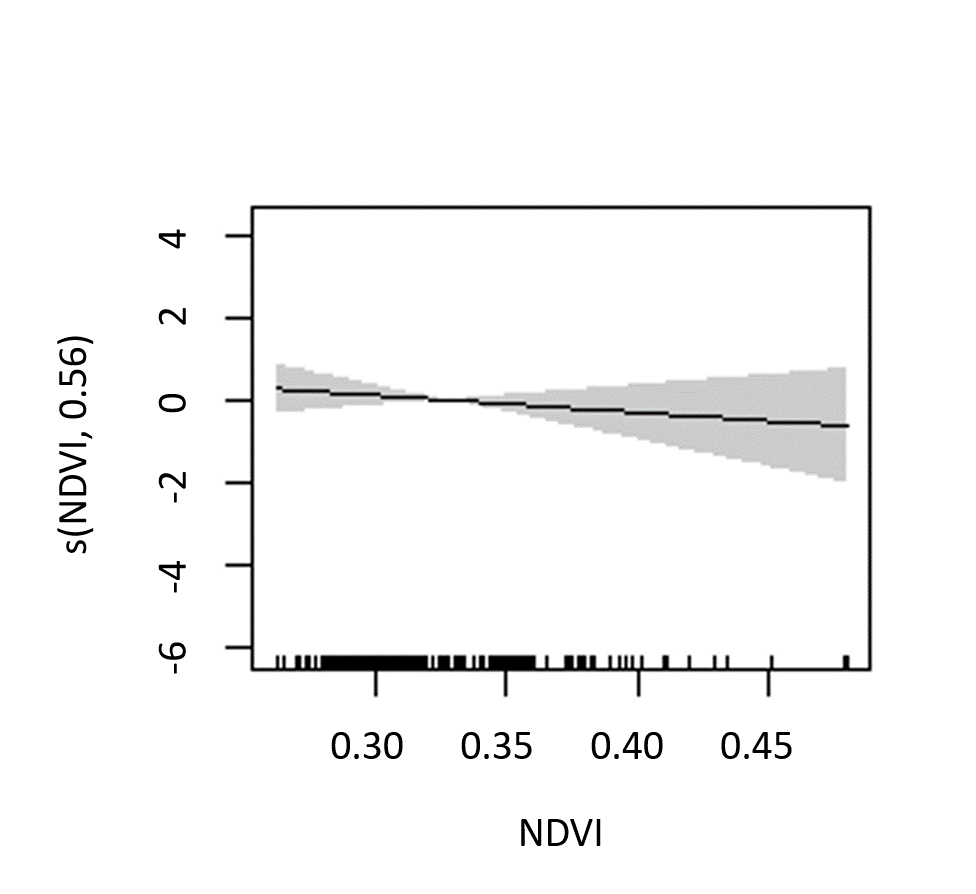


**b) Sambar**
