# Supplementary material for: Spatially explicit density and its determinants for Asiatic lions in the Gir forests
Source: PLoS One. 2020 Feb 19;15(2):e0228374. doi: 10.1371/journal.pone.0228374 (PMC7029878; doi:10.1371/journal.pone.0228374)
Supplement: S5 Fig — (DOCX) [file pone.0228374.s010.docx]

**Fig S5:** Scatter plots of covariates with SECR lion density and their statistics for a linear relationship**.**

**
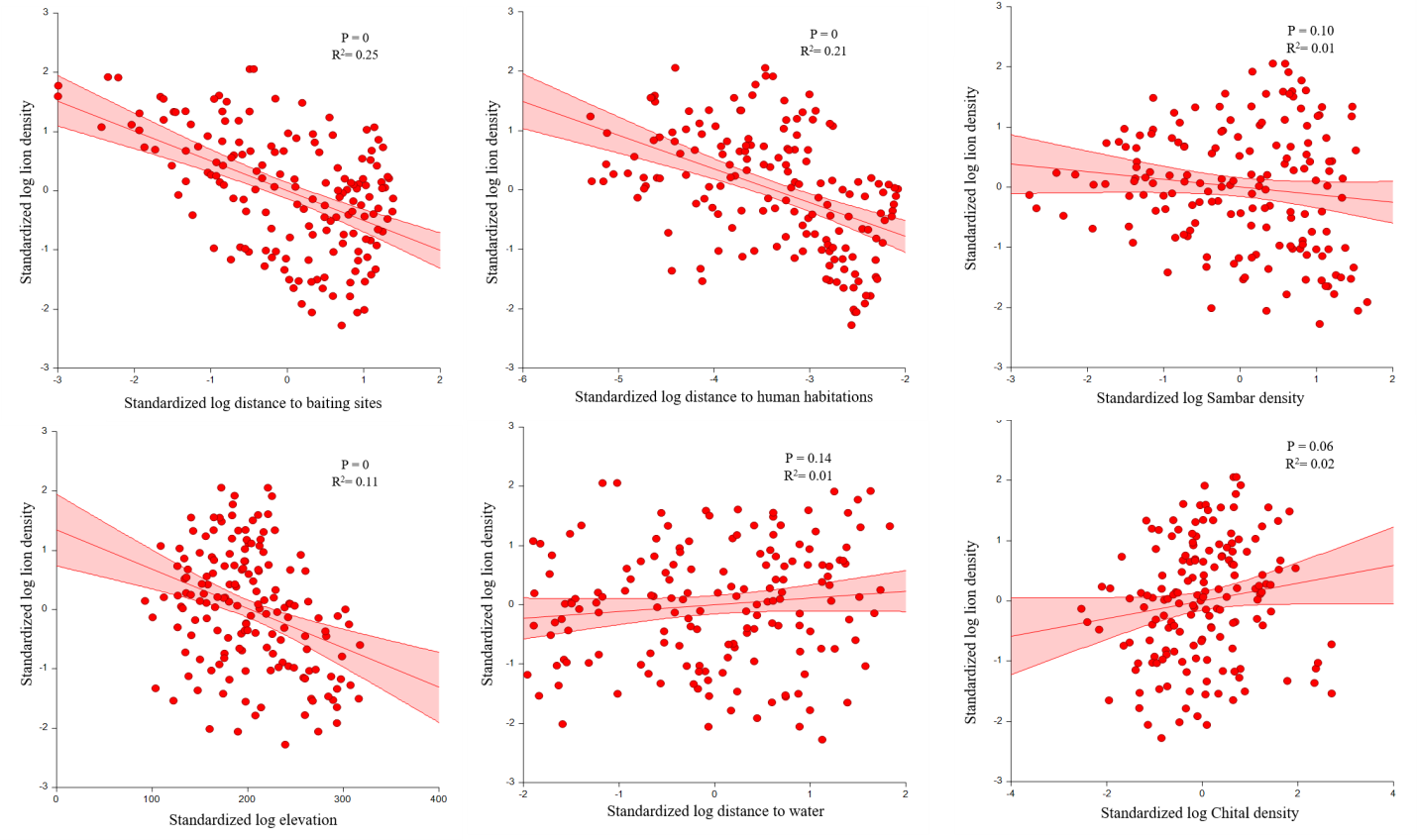
**
