# Supplementary material for: Spatially explicit density and its determinants for Asiatic lions in the Gir forests
Source: PLoS One. 2020 Feb 19;15(2):e0228374. doi: 10.1371/journal.pone.0228374 (PMC7029878; doi:10.1371/journal.pone.0228374)
Supplement: S6 Fig — (DOCX) [file pone.0228374.s011.docx]

**Fig S6:** Residual plots and qq plots for testing normality of the residuals of the best fit model explaining lion density as a function of distance to baiting sites, elevation, and distance to human habitation.

KS test failed to reject normality of residuals (D = 0.103, p-value = 0.063)

1. Sambar:

**
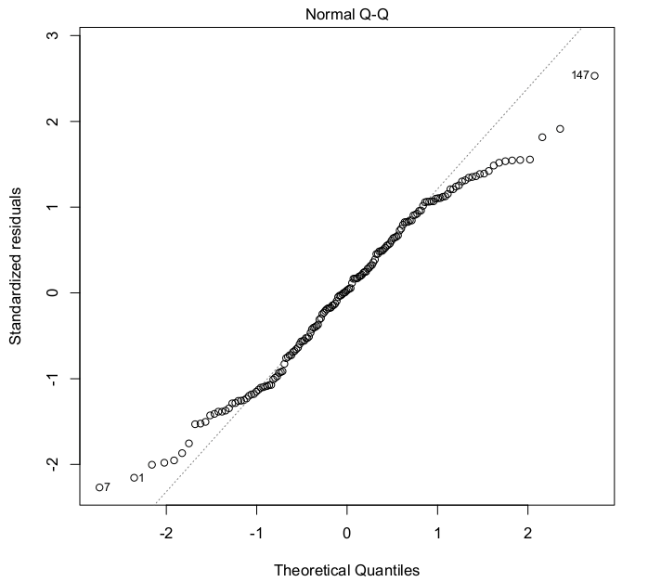

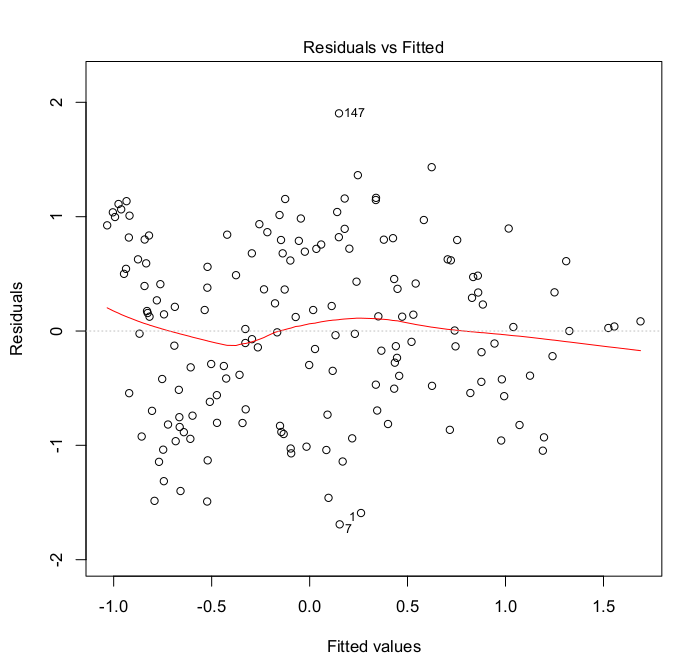
**
